# Description of Two New Species of *Stauroneis* Ehrenberg (Naviculales, Bacillariophyceae) from the Russian Far East Using an Integrative Approach

**DOI:** 10.3390/plants13152160

**Published:** 2024-08-05

**Authors:** Veronika B. Bagmet, Shamil R. Abdullin, Vyacheslav Yu. Nikulin, Arthur Yu. Nikulin, Tatiana Y. Gorpenchenko, Andrey A. Gontcharov

**Affiliations:** Federal Scientific Center of the East Asia Terrestrial Biodiversity, Far Eastern Branch of the Russian Academy of Sciences, 159, 100-Letia Vladivostoka Prospect, Vladivostok 690022, Russia; chara1989@yandex.ru (V.B.B.); nikulinvyacheslav@gmail.com (V.Y.N.); artyrozz@mail.ru (A.Y.N.); gorpenchenko@biosoil.ru (T.Y.G.); gontcharov@biosoil.ru (A.A.G.)

**Keywords:** integrative approach, isogamy, morphological characteristics, new species, nonaquatic habitats, *rbc*L gene, *Stauroneis*, temperate monsoon climate zone

## Abstract

*Stauroneis* (Naviculales, Bacillariaceae) are widespread, mostly in fresh-water habitats, and account for 343 species. They are described mainly on the basis of morphology and morphometric traits. These characteristics vary during life cycles and may overlap between species, making their identification difficult. We isolated two strains of naviculoid diatoms and examined them using an integrative approach (phylogenetic, morphological, ultrastructural data, and life cycle). Phylogenetic analyses based on chloroplast *rbc*L gene data showed affinity of the new strains to the genus *Stauroneis*. Our algae share morphological features typical of *Stauroneis* but differ from similar species in minimal valve length measurements, valve apex shape, and minimal number of striae in 10 μm. Two strains are distinct from each other in maximal valve length and width, partially valve shape, the number of areolae in 10 μm, and cingulum structure. It was revealed that the strains reproduce via isogamy. Three species delimitation methods (ASAP, PTP, and GMYC) also confirmed that the two closely related new strains represent distinct species. Based on molecular data and phenotypic traits examined within the framework of an integrative approach, we describe two new isolates as *Stauroneis urbani* sp. nov. and *Stauroneis edaphica* sp. nov.

## 1. Introduction

Species of *Stauroneis* Ehrenberg are naviculoid diatoms belonging to the order Naviculales of the class Bacillariophyceae. They are characterized by lanceolate or elliptical valves with single-row striae, containing small round poroids; and, the hyaline central area known as stauros, which is a characteristic trait of this genus. *Stauroneis* species usually inhabit freshwater and are mainly epipelic, although a few are aerophytic and grow on mosses and soil surface [1,2,3,4,5]. According to the AlgaeBase [6], the genus *Stauroneis* is widely distributed over the world and accounts for 343 species.

Some morphological characters in algae, including diatoms, are of polymorphic nature. Therefore, species delimitation based on morphology alone can lead to errors in taxonomic identification [7]. The sizes and shapes of the valves undergo alterations throughout the life cycle of diatoms, and teratological forms appear [1,8,9,10]. Consequently, an integrative approach, including examination of morphological and morphometric characteristic ranges, genetic data analysis, and life cycle with the sexual process, is important for diatom taxonomy. There is little molecular genetic data on the genus members, despite its constantly growing number of species. In the GenBank database (https://www.ncbi.nlm.nih.gov; accessed on 2 April 2024), *Stauroneis* is associated with 60 accessions of chloroplast *rbc*L (32) and 18S rRNA genes (28), representing 14 species, and 4 taxa identified only to the genus level. So far, the sexual process was examined only in *Stauroneis anceps* Ehrenberg, *S. gracilior* E.Reichardt, *S. legumen* (Ehrenberg) Kützing, *S. phoenicenteron* (Nitzsch) Ehrenberg, and *S. siberica* (Grunow) Lange-Bertalot et Krammer [11,12,13]. 

In this contribution, we provide a description of two newly determined species of naviculoid diatoms isolated during a study of algal diversity in aerophytic habitats of architectural structures of urban ecosystems and soils in the temperate monsoon climate zone in the south of the Russian Far East: *Stauroneis urbani* sp. nov. and *Stauroneis edaphica* sp. nov. using the integrative approach.

## 2. Results

### 2.1. Taxonomic Determinations

**Figure 1:**
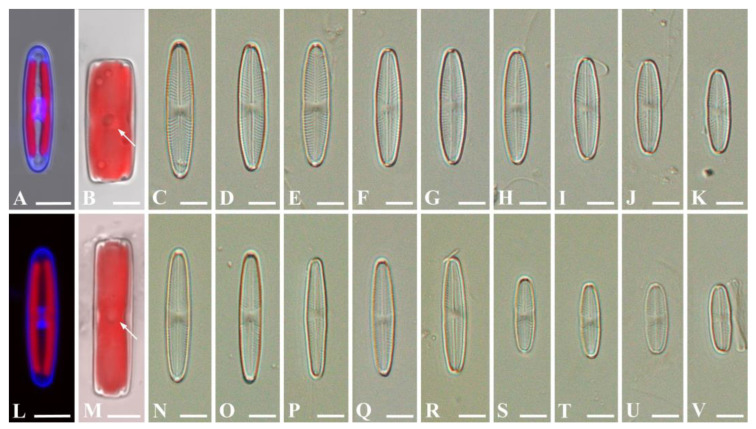
*Stauroneis urbani* sp. nov. (**A**–**K**) and *Stauroneis edaphica* sp. nov. (**L**–**V**). (**A**,**L**)—valve with two chloroplasts and nucleus between them stained with DAPI; (**B**,**M**)—valve in the girdle view with chloroplast and round pyrenoid (white arrow); (**C**–**K**,**N**–**V**)—valves of vegetative cells. Scale bar: 5 μm. Light microscopy (**C**–**K**,**N**–**V**). Confocal laser scanning microscopy (**A**,**B**,**L**,**M**).

**Figure 2:**
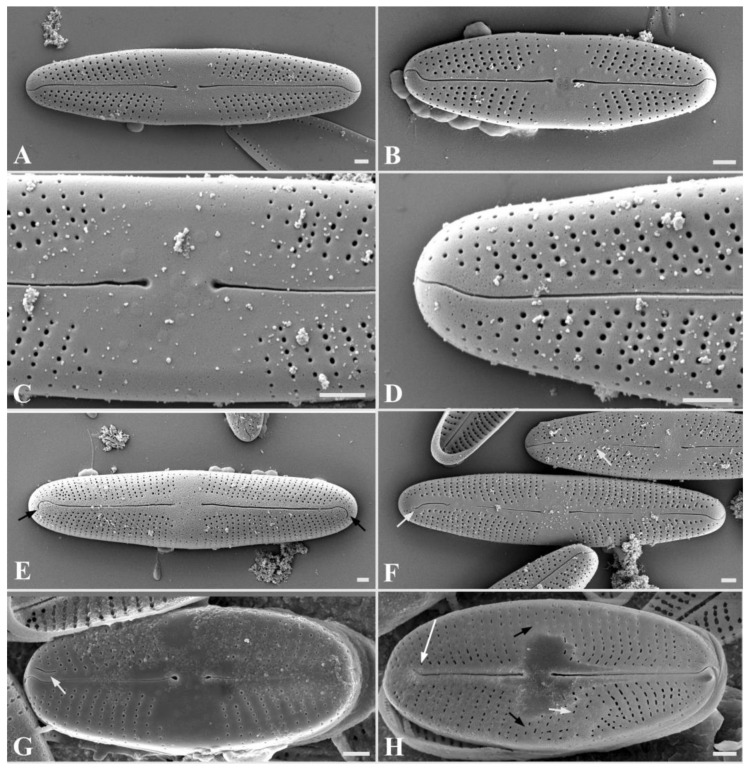
*Stauroneis urbani* sp. nov. Scanning electron microscopy (SEM), external view (**A**–**E**): (**A**)—general view of large vegetative cell; (**B**)—general view of small vegetative cell; (**C**)—central area with proximal raphe ends; (**D**)—distal raphe end; (**E**)—primary initial cells with hook-shaped distal ends, not extending to the surface of the mantle (white arrows). SEM, teratological forms (**F**–**H**): (**F**)—initial cell with disruption of raphe branch (white arrows); (**G**)—teratological valve with double branch of raphe (white arrow); (**H**)—teratological valve with a shortened branch of raphe (the distal end is on the surface of the valve, long white arrow), bordering of the central area (small black arrows), randomly located striae (small white arrow). Scale bar: 1 μm.

**Figure 3:**
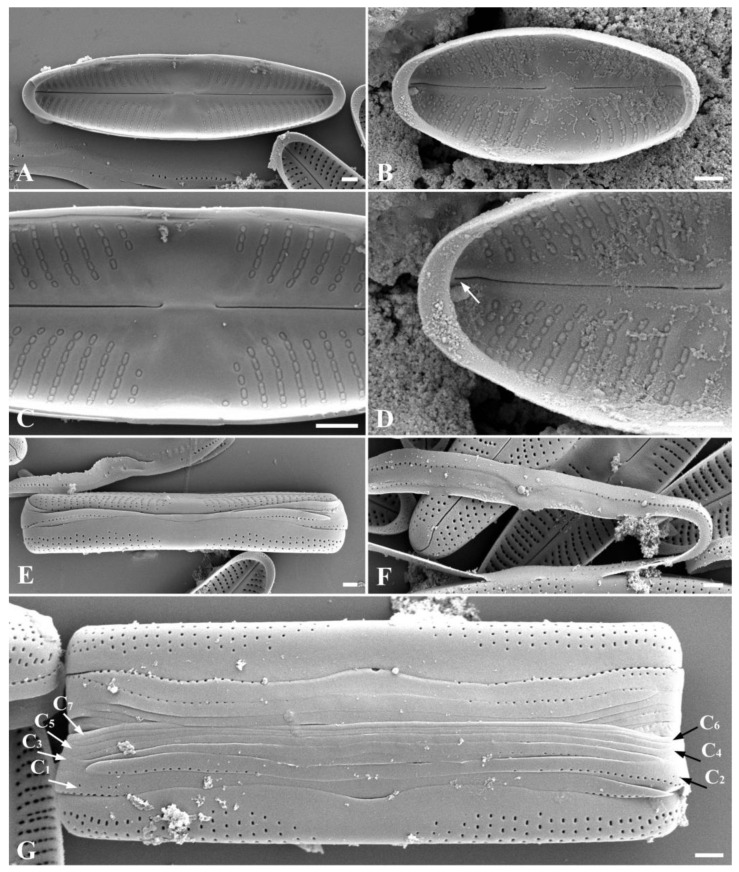
*Stauroneis urbani* sp. nov. SEM, internal view (**A**–**D**): (**A**)—general view of large vegetative cell; (**B**)—general view of small vegetative cell; (**C**)—central area with proximal raphe ends; (**D**)—distal raphe end terminated small helictoglossae (white arrow). SEM, structure of cingulum (**E**–**G**): (**E**)—immature cingulum consisting of five copulae; (**F**)—structure of valvocopula; (**G**)—mature cingulum consisting of seven copulae ((**C_1_**–**C_7_**); white and black arrows). Scale bar: 1 µm.

*Stauroneis urbani* Bagmet, Abdullin, A. Nikulin, V. Nikulin, and Gontcharov sp. nov. Figure 1A–K, Figure 2A–H and Figure 3A–G.

Holotype: Exsiccatum number VLA-CA-1856, a dried biomass of a clonal strain, was deposited in the Herbarium, Federal Scientific Center of East Asian Terrestrial Biodiversity, Vladivostok, Russia. Gene sequence: DNA sequence obtained from a clonal strain of *Stauroneis urbani* was deposited in the GenBank under accession no. PP708879.

Type locality: Vladivostok city, Primorsky Territory, Russia (43°07′29.8″ N 131°53′28.1″ E), concrete biofouling on the architectural structure.

Etymology: The species epithet is a reference to the fact that it is derived from an urban ecosystem (from the Latin “urbanus”).

Distribution: So far, it is only known to be from Vladivostok city.

Comment: It differs from similar *Stauroneis* species in the following set of morphological characters: minimal valve length, valve apices shape, and minimal number of striae in 10 μm. It is distinct from *S. edaphica* in maximal valve length and width, partial valve shape, number of areolae in 10 μm, and cingulum structure; and differences in the chloroplast *rbc*L gene sequence.

Description of *Stauroneis urbani*:

Light microscopy (LM) (Figure 1A–K). Live cells are solitary and actively moving. The valves are linear-lanceolate, lanceolate to almost elliptical, with widely rounded apices, slightly narrowed in the central part (Figure 1C–K). Valve dimensions (*n* = 40): length 9.7–30.1 μm; width 3.8–6.4 μm. Striae radiate, 19–27 in 10 μm.

Confocal laser scanning microscopy (CLSM) (Figure 1A,B). Frustules rectangular in girdle view (Figure 1B). Cell with two chloroplasts, on either side of the apical plane during interphase (Figure 1A). Each chloroplast contains a pyrenoid, which is central, round, and invaginated (Figure 1B). The interphase nucleus is oval in shape, occupies a central position, and is clearly visible from the valve side (Figure 1A).

SEM, external view (Figure 2A–D). Axial area narrow, expanded to the valve center (Figure 2A,B). Central area is broad, bow-tie-shaped (Figure 2C). Raphe is filiform (Figure 2A,B). Proximal raphe ends straight, teardrop-shaped (Figure 2C). Distal raphe ends are hook-shaped, extending onto the surface of the mantle (Figure 2D). The areolae are round, also extending onto the surface of the mantle (Figure 2D and Figure 3E,G), 27–37 in 10 μm. 

SEM, internal view (Figure 3A–D): Axial area narrow, expanded to the valve center (Figure 3A,B). Central area is bow-tie-shaped, stauros is absent (Figure 3C). Raphe is filiform (Figure 3A). Proximal raphe ends curved (Figure 3C). Distal raphe ends terminate as small helictoglossae (Figure 3D). Striae radiate, consist of elongated oval areolae, occluded by hymens, which are located at a small distance from each other and have a round or oval shape (Figure 3A–D). Pseudosepta clear, covering the distal raphe ends (Figure 3D). 

SEM, cingulum (Figure 3E–G): Striae extend onto the surface of the mantle (Figure 3E,G). Immature cingulum consists of five copulae (Figure 3E), mature cingulum of seven copulae (Figure 3G). The valvocopula is perforated with a single row of pores; towards the end of the valve, the row of pores becomes double (Figure 3F). The remaining copulae are morphologically similar; each copula has one row of rounded pores. There is no perforation in the central part of all copulae (Figure 3G).

Teratological forms (Figure 2F–H): Teratological forms were observed both in young initial cells (Figure 2F) and in small vegetative cells with a length of less than 13 μm (Figure 2G,H). They were represented by an atypical raphe (shortened raphe branches, double raphe, disruption of raphe) and striae aberration (Figure 2F–H).

**Figure 4:**
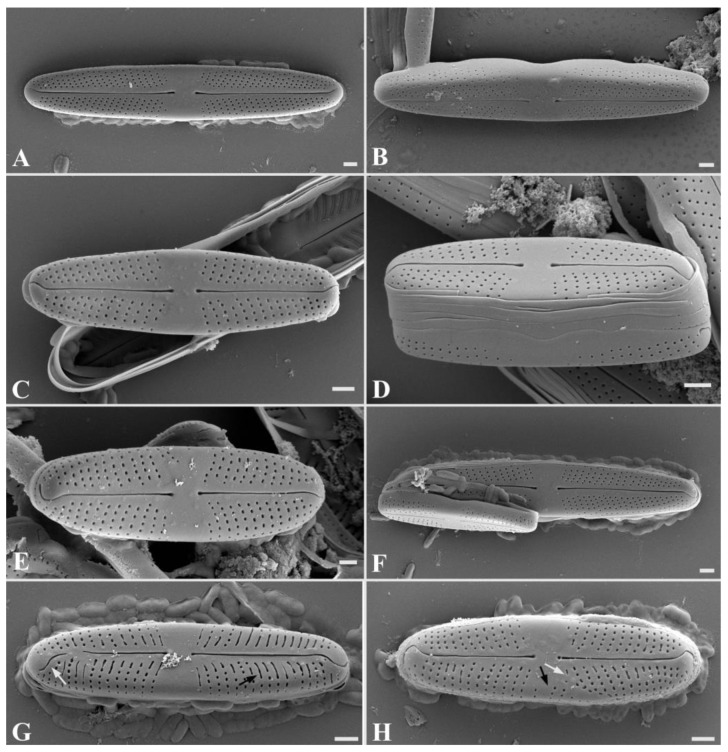
*Stauroneis edaphica* sp. nov. SEM, external view (**A**–**F**): (**A**,**B**)—general view of large vegetative cell; (**C**–**E**)—general view of small vegetative cell; (**F**)—initial cell adjacent to the parent valve. SEM, teratological forms (**G**,**H**): (**G**)—teratological valve with elongated slit-like areolae (black arrow) and disruption of the raphe branch (white arrow); (**H**)—teratological valve with striae in the central area of the valve (black arrow) and striae aberration (white arrow). Scale bar: 1 μm.

**Figure 5:**
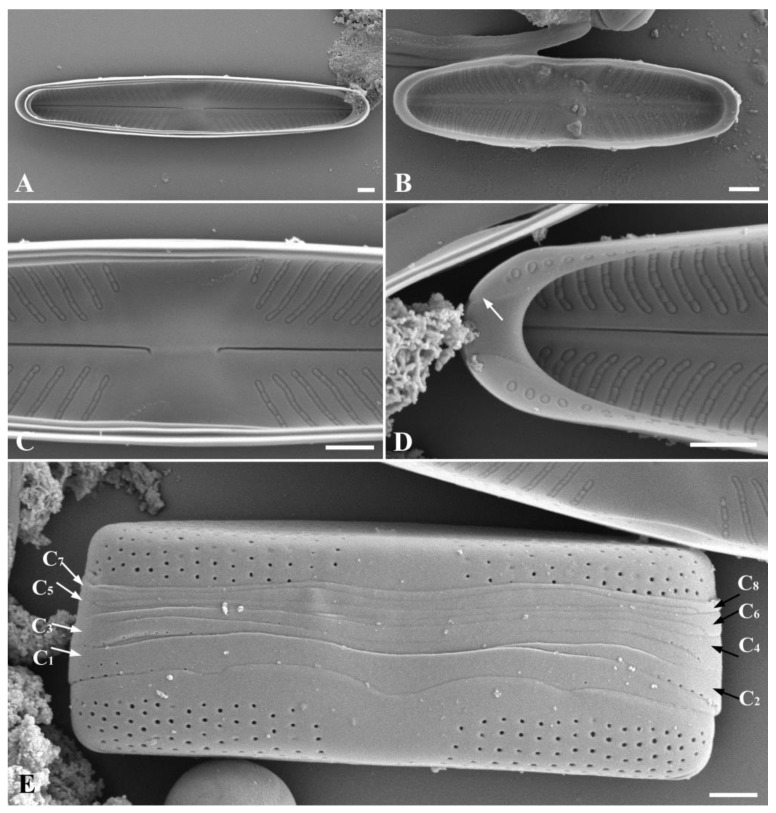
*Stauroneis edaphica* sp. nov. SEM, internal view (**A**–**D**): (**A**)—general view of large vegetative cell; (**B**)—general view of small vegetative cell; (**C**)—central area with proximal raphe ends; (**D**)—pseudosepta covering the distal raphe end, open valvocopula (white arrow). SEM, structure of cingulum (**E**): (**E**)—mature cingulum consisting of eight copulae ((**C_1_**–**C_8_**); white arrows). Scale bar: 1 µm.

*Stauroneis edaphica* Bagmet, Abdullin, A. Nikulin, V. Nikulin, and Gontcharov sp. nov. Figure 1L–V, Figure 4A–H and Figure 5A–E.

Holotype: Exsiccatum number VLA-CA-1702, a dried biomass of a clonal strain, was deposited in the Herbarium, Federal Scientific Center of East Asian Terrestrial Biodiversity, Vladivostok, Russia. Gene sequence: DNA sequence obtained from a clonal strain of *Stauroneis edaphica* was deposited in the GenBank under accession no. PP708880.

Type locality: broad-leaved forest on the slope of Mount Sestra, Primorsky Territory, Russia (42°49′18.5″ N 132°59′40.8″ E), soil.

Etymology: The species epithet is a reference to the fact that it is a soil alga (from the Greek “edaphos”).

Distribution: So far, it is only known to be from the slope of Mount Sestra.

Comment: It differs from other similar *Stauroneis* species based on the following set of morphological characters: minimal valve length, valve apex shape and minimal number of striae in 10 μm. Differs from *Stauroneis urbani* in maximal valve length and width, partial valve shape, number of areolae in 10 μm, and cingulum structure, as well as differences in the chloroplast *rbc*L gene sequence.

Description of *Stauroneis edaphica*:

LM (Figure 1N–V): Live cells are solitary and actively moving. Valves from rhombic-lanceolate, lanceolate, to elliptic-lanceolate with widely rounded ends (Figure 1N–V). Only small valves slightly narrowed in central part (Figure 1S–V). Valve dimensions (*n* = 40): length 12.7–25.6 μm; width 3.3–4.6 μm. Striae radiate, 19–29 in 10 μm.

CLSM (Figure 1L–M): Frustules rectangular in girdle view (Figure 1M). Cell contains two chloroplasts, one on either side of the apical plane during interphase (Figure 1L). Each chloroplast contains a pyrenoid, which is central, round, and invaginated (Figure 1M). The interphase nucleus is oval, occupies a central position, and is clearly visible from the valve side (Figure 1L).

SEM, external view (Figure 4A–F): Axial area narrow, expanded to the valve center (Figure 4A–F). Central area is broad, bow-tie-shaped (Figure 4A–F). Raphe is filiform (Figure 4A–F). Proximal raphe ends straight, teardrop-shaped (Figure 4A–F). Distal raphe ends are hook-shaped, extending onto the surface of the mantle (Figure 4A–F). The areolae are round, also extending onto the surface of the mantle (Figure 4D), 34–41 in 10 μm.

SEM, internal view (Figure 5A–D): Axial area narrow, expanded to the valve center (Figure 5A,B). Central area is bow-tie-shaped, stauros is absent (Figure 5C). Raphe is filiform (Figure 5A,B). Proximal raphe ends curved (Figure 5C). Striae radiate, consist of elongated, tightly adjacent-to-each-other oval areolae, occluded by hymenes (Figure 5A–D). Occluded hymenes strongly pressed against each other, elongated, and have a rectangular shape (Figure 5C,D). Pseudosepta clear, covering the distal raphe ends (Figure 5D).

SEM, cingulum (Figure 5E): Striae extend onto the surface of the mantle (Figure 5E). A mature cingulum consists of eight open copulae (Figure 5E). The valvocopula is perforated with a single row of pores; towards the end of the valve, the row of pores becomes double. The remaining copulae are morphologically similar; each copula has one row of rounded pores. There is no perforation in the central part of all copulae (Figure 5E).

Teratological forms (Figure 4G,H): Teratological forms were observed in small vegetative cells with a length less than 14 μm (Figure 4G,H). They were represented by an atypical raphe (disruption of raphe), striae aberration, elongated slit-like areolae, and striae in the central area of the valve (Figure 4G,H).

### 2.2. Phylogenetic Status

**Figure 6:**
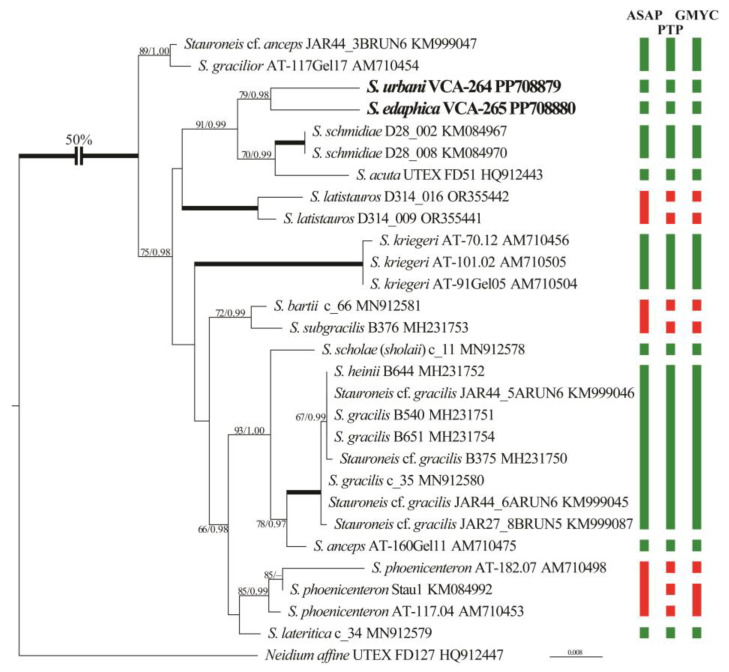
ML phylogenetic tree of the genus *Stauroneis* (TIM2+I+G model) showing position of new strains based on partial *rbc*L gene sequence data (29 sequences, 1259 aligned positions). Supports [(BP) > 50% and (PP) > 0.95: ML/BI] are provided above/below the branches. New strains and branches with 100% BP and 1.00 PP are shown in boldface. Scale bar: substitutions per nucleotide position. Rectangles indicate results of species delimitation using the ASAP, PTP, and GMYC methods (clusters of species-level sequences). Green rectangles represent identical clusters across all three methods, while red rectangles indicate disagreement in delimitation results.

Phylogenetic analysis of 28 *Stauroneis rbc*L sequences showed that the new strains form a moderately supported clade (79/0.98; Figure 6) and are characterized by long individual branches. Clade (70/0.99) were composed of *S. schmidiae* (two sequences), and *S. acuta* was in the sister position (91/0.99). The overall branching pattern within the genus was weakly resolved.

### 2.3. Molecular Delimitation of Species

Intraspecific values of *p*-distances among *Stauroneis* sequences did not exceed 0.84%, while the interspecific values were greater (Table S1). The only exceptions were *S. gracilis/S. heinii* and *S. subgracilis/S. bartii*, whose interspecific distances were below this threshold. Despite close affinity, new strains differed from each other by 2.31%.

Three molecular species delimitation methods (ASAP, PTP, and GMYC) were used to delimit species-level clusters within *Stauroneis*. Figure 6 compares the results based on *rbc*L sequences: three delimitation methods were almost congruent, delimiting from 13 to 17 species-level clusters. In most cases, individual clades or diverged accessions represented a single species according to these methods. The exceptions were the clades of *S. latistauros* and *S. bartii/subgracilis*, which were divided into two clusters by PTP and GMYC methods, and one by ASAP method. Additionally, the clade of *S. phoenicenteron* was distinguished into one to three species clusters by different methods. On the contrary, *Stauroneis* cf. *anceps* and *S. gracilior* are resolved into one species by all methods, as well as a group of species *S. heinii* and *S. gracilis*. Clades delimited as species are characterized by moderate or strong support (Figure 6). New strains were defined as two independent clusters by all methods.

### 2.4. Sexual Reproduction

**Figure 7:**
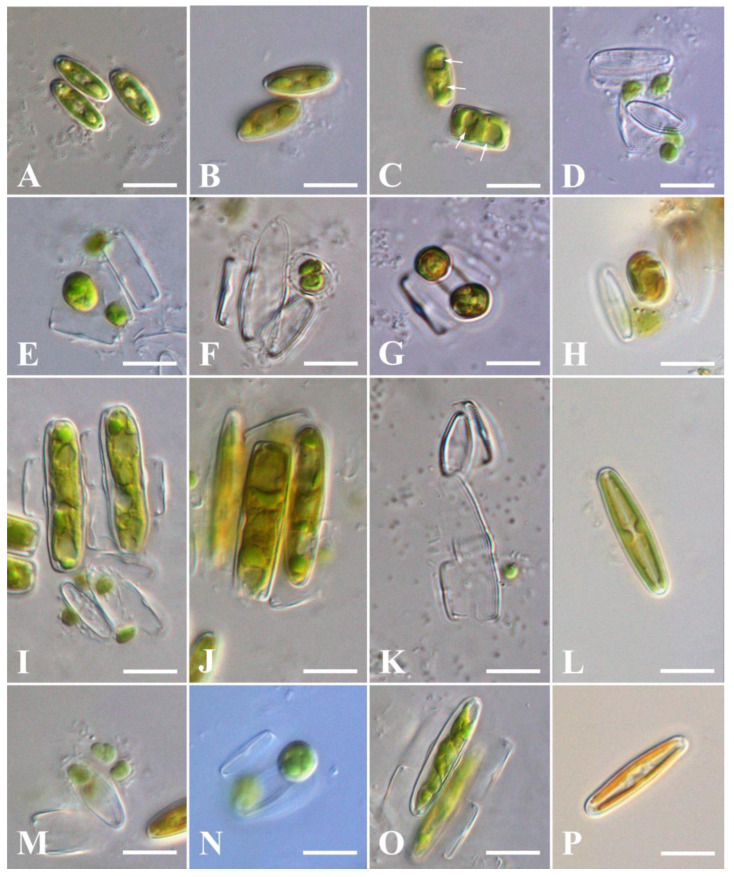
Sexual reproduction of *Stauroneis urbani* sp. nov. (**A**–**L**) and *Stauroneis edaphica* sp. nov. (**M**–**P**), LM: (**A**,**B**)—pairing cells; (**C**)—gametogenesis, forming of two haploid nuclei in cell (white arrows); (**D**,**M**)—releasing of gametes; (**E**)—syngamy, zygote forming; (**F**)—gametes fusion in incunabula; (**G**,**N**)—two zygotes; (**H**)—one zygote; (**I**,**J**,**O**)—auxospores; (**K**)—perizonium; (**L**,**P**)—initial cells. Scale bar: 10 µm.

Homothallic reproduction was observed in the monoclonal cultures of *S. urbani* and *S. edaphica* (Figure 7). There was no sexual reproduction between clones of these species.

Two cells align side-by-side (girdle–girdle), forming a gametangial pair (Figure 7A,B). Gametangia pairs actively and surround themselves with mucilage. At first, the gametangia are close to each other, but are not attached, and the mucilage sheath (copulation envelope) is narrow (Figure 7B). As meiosis proceeds, more mucilage is secreted, widening the sheath and also pushing the gametangia apart. Then, gametogenesis begins, including meiosis I and meiosis II, as a result of which two haploid nuclei are formed in each gametangium (Figure 7C), and the cell protoplast divides transapically and rounds off, resulting in the formation of two motile round gametes (Figure 7C,D). Mature gametes leave the gametangium and move outside, searching for a gamete from another gametangium (Figure 7D,M). However, both pairs of gametes do not always fuse. As a result of this syngamy (Figure 7E), one (Figure 7H) or two zygotes (Figure 7G) are formed. The zygote bipolarly expands and elongates parallel (Figure 7I,O) or perpendicular (Figure 7J) to the valves of the parent cells, turning into auxospores. This type of sexual reproduction could be classified as isogamy. It is designated as IC, according to Geitler [14]. This type of sexual reproduction was characteristic for both *S. urbani* and *S. edaphica*. 

In *S. urbani* we observed a thin silicate layer—incunabula (Figure 7F), surrounding the zygote, and a perizonium (Figure 7K), enclosing the auxospore. After maturation, the initial cell (Figure 7L,P) escaped from the perizonium and went outside, where it began to actively divide vegetatively.

Initiation of sexual reproduction in *S. urbani* began when the parental cells reached a valve length of 10.5–13.8 µm. After sexual reproduction, the length of the initial cells increased to 24.6–30.1 μm (Figure 7L). The primary initial cells were morphologically different from the vegetative ones: they were more rounded, without a clear division into the mantle and valve (Figure 2E) and possessed morphological teratologies such as the disruption of the raphe (Figure 2F) and the raphe with hook-shaped distal ends not extending to the surface of the mantle (Figure 2F). However, all these teratologies disappeared after several subsequent vegetative divisions.

In *S. edaphica*, initiation of sexual reproduction began when the parental cells reached a valve length of 12.3–14.2 µm. After sexual reproduction, the length of the initial cells increased to 22.9–26.2 μm (Figure 7P).

## 3. Discussion

### 3.1. Morphological Analysis

Van de Vijver and colleagues [2] identified two major groups in the genus *Stauroneis*, distinguished by the presence or absence of pseudosepta. These groups were further divided into seven subgroups based on characters such as valve length and width, valve ends, central raphe endings, valve shape, striation patterns and presence/absence of marginal or raphe ridges. Species without pseudosepta were classified into (1) very large valves (length > 75 μm): *S. gracilis*/*heinii*-like; (2) smaller valves (length > 40 μm and < 80 μm): *S. subgracilis*/*anceps*-like and *S. gracilior*-like; (3) very small valves (length < 40 μm): *S. kriegeri*/*agrestis*-like. Species having pseudosepta were classified as (4) *S. hyperborea*-like; (5) *S. obtusa*-like; (6) *S. sagitta*-like; and (7) small-celled taxa with pseudosepta. Bahls [3] divided North American *Stauroneis* into seven groups based on a combination of five morphological features (shape and position of areolae; the presence or absence of pseudosepta; valve wide; and end shapes of valves): (1) *S. phoenicenteron* group; (2) *S. gracilis* group; (3) *S. anceps* group; (4) *S. americana* group; (5) *S. siberica* group; (6) *S. agrestis* group; and (7) *S. stodderi* group. These two classifications are similar, although Bahls [3] analyzed only N. American species of *Stauroneis*. 

The morphological features of two new species fit characteristics of the group 7 (small-celled taxa with pseudosepta) and one species of group 6 (*S. sagitta*-like with pseudosepta), according to the classification of Van de Vijver et al. [2], and one species of the *S. americana* group, according to the classification of Bahls [3]. Therefore, we compared morphologies of *S. urbani* and *S. edaphica* with those of these groups’ members, as well as with phylogenetically related *S. schmidiae*. Species with large valves (maximal length of ≥32 μm), including *S. acuta* that also showed some affinity according to the phylogenetic data, were excluded from the analysis as clearly different from *S. urbani* and *S. edaphica* (Table S2).

Despite having some similar appearance, *S. urbani* and *S. edaphica* can be distinguished from most other species by their minimal valve length, valve apices, and minimal number of striae in 10 μm. *Stauroneis edaphica* is similar only to *S. thermicoloides* Van de Vijver and Lange-Bertalot, and *S. microproducta* Van de Vijver and Lange-Bertalot, in the minimal valve length. However, they differ from our species in the valve shape and apices (Table S2). Both our species are similar only to *S. schmidiae* in shape of the valve apices and minimal number of striae in 10 μm. *Stauroneis urbani* and *S. edaphica* differ from *S. schmidiae* in minimal valve length, maximal number of striae and areolae in 10 μm (Table S2).

*Stauroneis urbani* and *S. edaphica* differ from each other in maximal valve length and width, partially valve shape, in the number of areolae in 10 μm, and in structure of cingulum (Table S2, Figure 3E,G and Figure 5E). These species also differ in structure of occluded hymenes: in *S. urbani*, they are located at a small distance from each other and have a round or oval shape (Figure 3A–D); in *S. edaphica,* they are strongly pressed against each other, elongated, and have a rectangular shape (Figure 5C,D). The first form of occluded hymenes is relatively uncommon: *S. kriegeri* R.M.Patrick [2], *S. miyakoensis* A. Tuji [15], *S. saprophila* M. Rybak, T. Noga and Ector [16]. The second form of occluded hymenes is observed in a large number of *Stauroneis* species, for example: *S. anceps* Ehrenberg [2], *S. gracilis* Ehrenberg [7], *S. lateritica* Wadmare, Kociolek and B. Karthick [7], *S. schmidiae* R. Jahn and N. Abarca [17], *S. sikkimensis* N. Wadmare, S. Roy, Kociolek and B. Karthick [18], etc. 

A typical feature of the genus *Stauroneis* is a stauros [1]. However, it can be narrow, linear, or widest at the center in some species of genus (*S. smithii* Grunow) [19], rectangular, slightly widened with (4–7) shortened striae (*S. bartii* Wadmare, Kociolek and B. Karthick) [7], bow-tie shaped (*S. lateritica* Wadmare, Kociolek, and B. Karthick) [7], narrow rectangular (*S. thermicola* (J. B. Petersen) J. W. G. Lund) [20], etc. In our two species, the stauros is broad and bow-tie shaped. Thus, the size and shape of the stauros can vary among different species of *Stauroneis*.

Teratological forms in our species were observed either in initial cells or in very small cells. It is known that morphology of initial cells (e.g., rounded shape, underdeveloped raphe, different number of striae or/and areolae, etc.) differs from vegetative ones. All these differences disappear after several vegetative divisions [10,21]. The appearance of teratological forms in small cell valves may be caused by their long-term cultivation; such morphological changes are observed in many genera of diatoms: *Diatoma* [22,23]; *Cocconeis*, *Cyclotella*, *Cymbella*, *Encyonema*, *Fragilaria*, *Gomphonema*, *Mayamaea*, *Reimeria*, *Ulnaria* [24]; *Nitzschia* [9], etc.

There is little information on the structure of the cingulum in the species of the genus *Stauroneis*. It is assumed that there is one type of copulae structure: perforated by one row of pores. This type is characteristic for our two taxa as well. Two types of valve margins can be distinguished: straight, as in *S. acidojarensis* Zidarova, Kopalová and Van de Vijver [25], *S. delicata* Zidarova, Kopalová and Van de Vijver [25], *S. lateritica* Wadmare, Kociolek and B. Karthick [7], *S. lepchae* N. Wadmare, S. Roy, Kociolek and B. Karthick [18], *S. smithii* Grunow [19], and concave in the central part, as in *S. australobtusa* Zidarova, Kopalová and Van de Vijver [25], *S. sikkimensis* N. Wadmare, S. Roy, Kociolek and B. Karthick [18], and in our species *S. urbani* and *S. edaphica*.

To date, a detailed description of pyrenoid structure is known only for *S. phoenicenteron*. In this species, each chloroplast contains several pyrenoids (up to 9), which can be detected in valve view as slight inward thickenings of the chloroplast [11]. In *S. urbani* and *S. edaphica,* each chloroplast contains a single invaginated pyrenoid, which is very poorly visualized and can only be seen from the valve view. The interphase nucleus is oval in our species, but round in *S. phoenicenteron* [11].

### 3.2. Phylogeny and Species Delimitation

Relationships between *Stauroneis* species established in our analyses are generally consistent with previous studies [7,26]. Chloroplast-encoded *rbc*L gene sequence comparisons (Figure 6) placed the new species into the highly supported clade as a sister to *S. schmidiae* and *S. acuta*. Given the significant morphological differences between our species, this relationship may be due to incomplete taxon sampling.

Several studies demonstrated the ability of *rbc*L and other markers to distinguish closely related diatom species in combination with GMYC, ABGD, ASAP, and PTP methods [27,28,29]. In most cases, lineages identified by the species delimitation algorithms were congruent with the morphology-based species. Our analyses identified 13 to 17 species-level clusters in the dataset (Figure 6), where the studied strains were resolved as two separate clusters. The sequences of *S. latistauros* and *S. phoenicenteron*, despite their taxonomic assignment, were separated into one, two, or three clusters. This fact may be explained by the high value of intraspecific distances. The boundary between intraspecific and interspecific polymorphism for the studied species is approximately 0.84% (Table S1). Similar threshold values (0.8%) were obtained for the genera *Fragilaria* and *Ulnaria* [28].

Probably, the low values of the interspecific *p*-distances between the *Stauroneis* cf. *anceps*/*S. gracilior*, *S. gracilis*/*S. heinii*, and *S. subgracilis*/*S. bartii* influenced their grouping into common species clusters by the three methods and the ASAP method, respectively. 

As in many other diatom genera, only a few sequences of *Stauroneis* species are available in the GenBank database. Currently, *rbc*L sequence data is available for only 13 out of 343 species. The phylogenetic structure of the genus *Stauroneis* requires further scrutiny with more strains, sequences, and markers.

### 3.3. Sexual Reproduction

To date, sexual reproduction was examined in five species of the genus *Stauroneis*: *S. anceps*, *S. gracilior*, *S. legumen*, *S. phoenicenteron*, and *S. siberica* [11,12,13]. Like *S. urbani* and *S. edaphica*, these taxa are characterised by isogamy (designated IC, according to Geitler [14]). Other studied species of the family Stauroneidaceae were also isogamous (designated IC, according to Geitler [14]): of genera *Craticula* (*C. cuspidata* (Kutzing) D. G. Mann, *C. halophila* (Grunow) D. G. Mann, *C. importuna* (Hustedt) K. Bruder and Hinz, and *C. molestiformis* (Hustedt) Mayama [13,30]) and *Prestauroneis* (*P. protracta* (Grunow) Kulikovskiy and Glushchenko [31]). Perhaps isogamy is the only mode of sexual reproduction for the genus *Stauroneis* and family Stauroneidaceae.

## 4. Materials and Methods

### 4.1. Sampling and Culture Conditions

Naviculoid diatoms were isolated from concrete wall biofouling (Vladivostok city; 43°07′29.8″ N, 131°53′28.1″ E; 24 October 2019) and soil samples (forest, Mount Sestra, near Nakhodka city; 42°49′18.5″ N, 132°59′40.8″ E; 20 June 2022) collected in the temperate monsoon climate zone in the Primorsky Territory, Russia. The sampling was carried out in accordance with established protocols [32,33]. The clones were isolated via the micro-pipette method [34] and incubated into 40 mm Petri dishes with liquid nutrient medium Dm [35] under the following conditions: 20–22 °C, photon fluence 17.9–21.4 μmol photons·m^−2^s^−1^, and 16:8 h light–dark cycle. The clones were kept in the culture collection of the Laboratory of Botany in the Federal Scientific Center of East Asian Terrestrial Biodiversity, Russian Federation (clone numbers VCA-264 and VCA-265), and their dried biomasses were deposited in the Herbarium of the Federal Scientific Center of East Asian Terrestrial Biodiversity, Russia (exsiccatum numbers VLA-CA-1856 and VLA-CA-1702).

### 4.2. Microscopy

The morphology and morphometric traits of the diatom frustules were studied using an Olympus BX53 light microscope (LM) (Olympus Corporation, Tokyo, Japan) equipped with Nomarski DIC optics and an Olympus DP27 digital camera (Olympus Corporation, Tokyo, Japan), as well as a Merlin scanning electron microscope (SEM) (Carl Zeiss, Jena, Germany). Frustules were cleaned via oxidation with hydrogen peroxide, rinsed multiple times with distilled water, and then mounted in a Pleurax medium. Diatom material was dried onto brass stubs and coated with a gold–palladium (Au–Pd, 6:4) alloy to facilitate SEM. The morphometric data were analyzed using the software package Statistica 10.0 and Microsoft Office Excel 2007.

The fluorescence of chloroplasts in living cells was examined using two confocal laser scanning microscopes (CLSM), the LSM 510 META and the LSM 710 LIVE (Carl Zeiss, Jena, Germany), at the Instrumental Centre of Biotechnology and Gene Engineering of FSCEATB FEB RAS. To visualize the position of the nucleus, cells were stained with DAPI (Molecular Probes, Eugene, OR, USA) to visualize the position of the nucleus [36]. Files with the 3D-captured images were recorded and subsequently analyzed via LSM 510 Release v.4.2 and ZEN 2011 software.

### 4.3. Mating Experiments

Sexual reproduction in clones occurred during cultivation under the aforementioned conditions. Mixed cells were examined daily with an inverted light microscope CK30-F200 (Olympus Corporation, Tokyo, Japan) for three weeks in February–March 2022. Living cells, auxosporulation, and the stages of sexual reproduction were observed and described using LM following the methods described by Poulíčková and Mann [37] and Poulíčková et al. [36].

### 4.4. DNA Extraction, Amplification and Sequencing

For DNA analysis, cultures were harvested during the exponential growth phase period and concentrated via centrifugation. Subsequent procedures included DNA extraction, PCR amplification, and sequencing of the plastid-encoded *rbc*L gene, which were conducted in accordance with the methodology outlined by Bagmet et al. [38]. Sequences were assembled via the Staden Package v.1.4 [39]. Sequences of the partial *rbc*L gene were deposited in GenBank under accession numbers: PP708879 for *Stauroneis urbani* sp. nov. and PP708880 for *S. edaphica* sp. nov.

### 4.5. Alignment and Datasets

To clarify the phylogenetic position of the new strains, an alignment was constructed based on the dataset of Wadmare et al. [7], which included 28 taxa and 1259 bp of representatives of the *Stauroneis*. *Neidium affine* (Ehrenberg) Pfitzer HQ912447 (Neidiaceae) was chosen as an outgroup. The dataset was enriched by all sequences of *Stauroneis* available in the GenBank database. The sequences were aligned in the SeaView program [40].

### 4.6. Phylogenetic Analysis

Maximum likelihood (ML) analysis was conducted using PAUP 4.0b10 [41]. Bayesian inference (BI) was performed using MrBayes 3.1.2 [42]. In order to select the most appropriate DNA substitution model for the datasets, the Akaike information criterion (AIC; [43]) was employed with jModelTest 2.1.1 [44]. The TIM2+I+G model was identified as the optimal fit for the dataset. ML and BI analyses were conducted in accordance with the methodology outlined in Bagmet et al. [38]. The convergence of the stationary distribution was accessed by examining the ESS values, which exceeded 200, using Tracer v.1.7.1 [45]. The robustness of the ML trees was evaluated via bootstrap percentages (BP; [46]) and posterior probabilities (PP) in BI. BP < 50% and PP < 0.95 were not considered. ML-based bootstrap analysis was inferred using the web service RAxML v.7.7.1 (http://embnet.vital-it.ch/raxml-bb/; accessed on 10 April 2024; [47]).

### 4.7. Species Delimitation

The dataset was applied for species delineation using the Assemble Species by Automatic Partitioning (ASAP) method [48], accessed via the online service https://bioinfo.mnhn.fr/abi/public/asap/asapweb.html on 10 April 2024. The tree was reconstructed using the ML method, which was employed to delineate putative species by a maximum likelihood Poisson tree processes (PTP) model [49]. This was conducted using an online service https://species.h-its.org/ accessed on 10 April 2024. The generalized mixed Yule coalescent (GMYC) method [50] with the online service https://species.h-its.org/gmyc, accessed on 10 April 2024, was used to select clusters at the species level on the tree and to determine the species delimitation threshold. The ultrametric phylogenetic tree for the GMYC analysis was reconstructed by the Bayesian method in the BEAST v1.10.4 program [51] with an uncorrelated relaxed lognormal molecular clock. During phylogenetic reconstruction, the number of generations for Markov chains was set to ten million. Pairwise distances (*p*-distances) were estimated using MEGA 11 [52].

## Data Availability

The data presented in this study are available on request from the corresponding author. In addition, the data that support the findings of this study are openly available in GenBank.

## Supplementary Materials

The following supporting information can be downloaded at: https://www.mdpi.com/article/10.3390/plants13152160/s1; Table S1: Interspecific (lower diagonal) and intraspecific (diagonal) genetic *p*-distances (in %) estimated for *Stauroneis* species based on the *rbc*L gene sequences. Upper diagonal—standard error for interspecific *p*-distances. Table S2: Comparative analysis of morphology and morphometric traits in *Stauroneis urbani* and *Stauroneis edaphica* with morphologically similar species.
