# Supplementary material for: Description of Two New Species of Stauroneis Ehrenberg (Naviculales, Bacillariophyceae) from the Russian Far East Using an Integrative Approach
Source: Plants (Basel). 2024 Aug 5;13(15):2160. doi: 10.3390/plants13152160 (PMC11314381; doi:10.3390/plants13152160)
Supplement: Supplementary file 1 [file plants-13-02160-s001.zip › Table S1.pdf]

Table S1. Interspecific (lower diagonal) and intraspecific (diagonal) genetic *p*-distances (in %) estimated for *Stauroneis* species based on the *rbc*L gene sequences. Upper diagonal — standard error for interspecific *p*-distances.

	S. edaphica	***S. urbani***	*S. acuta*	*S. anceps*	*S. gracilis*	*S. gracilior*	*S. heinii*	*S. kriegeri*	*S. latistauros*	*S. phoenicenteron*	*S. schmidiae*	*S. sholaii*	*S. lateritica*	*S. bartii*	*S. subgracilis*
S. edaphica	n/c	0,41%	0,43%	0,68%	0,50%	0,80%	0,53%	0,82%	0,58%	0,68%	0,50%	0,46%	0,45%	0,43%	0,45%
S. urbani	2,31%	n/c	0,44%	0,72%	0,50%	0,75%	0,54%	0,79%	0,60%	0,68%	0,47%	0,47%	0,47%	0,47%	0,49%
S. acuta	2,71%	2,55%	n/c	0,72%	0,54%	0,79%	0,61%	0,83%	0,54%	0,69%	0,35%	0,52%	0,48%	0,44%	0,48%
S. anceps	3,79%	3,51%	3,65%	n/c	0,34%	0,64%	0,35%	0,64%	0,59%	0,49%	0,72%	0,42%	0,40%	0,52%	0,55%
S. gracilis	3,41%	3,33%	3,81%	0,94%	0,09%	0,63%	0,04%	0,73%	0,49%	0,46%	0,55%	0,33%	0,29%	0,39%	0,42%
S. gracilior	4,88%	4,02%	4,16%	3,30%	3,40%	n/c	0,64%	0,76%	0,64%	0,66%	0,75%	0,66%	0,58%	0,56%	0,66%
S. heinii	3,18%	3,18%	3,76%	0,98%	0,06%	3,44%	n/c	0,74%	0,48%	0,47%	0,56%	0,37%	0,33%	0,45%	0,49%
S. kriegeri	4,89%	4,41%	4,65%	3,34%	4,03%	3,64%	4,07%	0,05%	0,74%	0,66%	0,84%	0,72%	0,68%	0,69%	0,64%
S. latistauros	3,62%	3,52%	2,84%	3,16%	2,74%	3,52%	2,68%	4,09%	0,84%	0,53%	0,53%	0,48%	0,42%	0,42%	0,50%
S. phoenicenteron	4,22%	3,82%	3,87%	1,98%	2,19%	3,29%	2,23%	3,21%	3,03%	0,58%	0,71%	0,46%	0,34%	0,49%	0,53%
S. schmidiae	2,43%	2,11%	1,27%	3,79%	3,04%	4,02%	3,07%	4,79%	2,91%	3,96%	0,00%	0,56%	0,53%	0,49%	0,58%
S. sholaii	3,18%	2,87%	3,50%	1,26%	1,34%	3,30%	1,45%	3,63%	2,63%	2,01%	3,07%	n/c	0,33%	0,38%	0,41%
S. lateritica	2,87%	2,79%	2,95%	1,40%	1,18%	2,73%	1,25%	3,34%	2,05%	1,25%	2,64%	1,59%	n/c	0,30%	0,34%
S. bartii	2,55%	2,87%	2,55%	2,10%	2,38%	2,30%	2,51%	3,10%	2,05%	2,07%	2,11%	2,15%	1,35%	n/c	0,23%
S. subgracilis	2,79%	2,95%	2,87%	2,10%	2,54%	2,87%	2,70%	2,80%	2,68%	2,34%	2,75%	2,31%	1,51%	0,64%	n/c

Note: n/c – not calculated.
