# Supplementary material for: Description of Two New Species of Stauroneis Ehrenberg (Naviculales, Bacillariophyceae) from the Russian Far East Using an Integrative Approach
Source: Plants (Basel). 2024 Aug 5;13(15):2160. doi: 10.3390/plants13152160 (PMC11314381; doi:10.3390/plants13152160)
Supplement: Supplementary file 1 [file plants-13-02160-s001.zip › Table S2.pdf]

Table S2. Comparative analysis of morphology and morphometric traits in *Stauroneis urbani* and *Stauroneis edaphica* with morphologically similar species.

Diagnostic feature	*S. beyensii* Van de Vijver & Lange-Bertalot	*S. pseudo-schimanskii* Van de Vijver & Lange-Bertalot	*S. bertrandii* Van de Vijver & Lange-Bertalot	*S. leguminopsis* Lange-Bertalot & Krammer	*S. microproducta* Van de Vijver & Lange-Bertalot
Valve length, µm	27.0–30.0	24.0–30.0	22.0–25.0	20.0–30.0	14.0–17.0
Valve width, µm	6.5–7.5	6.0–7.0	4.5–6.0	4.0–6.0	3.5–4.5
Valve shape	lanceolate	elliptical-lanceolate, lanceolate, linear-lanceolate	linear-elliptic to linear	linear, triundulate	linear-lanceolate
Apices	shortly protracted, rostrate to subcapitate	shortly protracted, broadly subrostrate	abruptly protracted, rostrate to rostrate-subcapitate	abruptly protracted, elongated	clearly protracted, rostrate to capitate
Central area (Stauros)	narrow, rectangular, weakly expanded	distinctly expanded	narrow, slightly expanded	rectangular, very weakly expanded	narrow rectangular, not expanded
Proximal raphe ends, external view	straight	weakly curved	straight, pH-point-like	straight	straight
Striae in 10 µm	28–30	23–24	26–30	21–25	36
Areolae in 10 µm	ca. 38–40	20–25	36	36–42	>35
Striation pattern	moderately radiate throughout	moderately radiate	moderately radiate, more radiate to apices	moderately radiate throughout	radiate throughout
References			Van de Vijver et al., 2004		

Table S2. Comparative analysis of morphology and morphometric traits in *Stauroneis urbani* and *Stauroneis edaphica* with morphologically similar species (continued).

Diagnostic feature	*S. thermicoloides* Van de Vijver & Lange-Bertalot	*S. smithii* Grunow	*S. schmidiae* R.Jahn & N.Abarca	*S. urbani* sp. nov.	*S. edaphica* sp. nov.
Valve length, µm	13.0–17.0	18.0–30.0	27.0–28.2	9.7–30.1	12.7–25.6
Valve width, µm	2.5–3.5	4.0–9.0	5.5–6.0	3.8–6.4	3.3–4.6
Valve shape	linear-lanceolate	elliptical-lanceolate to lanceolate, triundulate margins	linear-lanceolate	linear-lanceolate, lanceolate to almost elliptical	rhombic-lanceolate, lanceolate to elliptic-lanceolate
Apices	clearly protracted, rostrate to subcapitate	narrowly rostrate	very slightly rounded non-protracted	widely rounded	widely rounded
Central area (Stauros)	broad, strongly expanded	narrow, linear, widest at the center	broad, bow-tie shaped	broad, bow-tie shaped	broad, bow-tie shaped
Proximal raphe ends, external view	straight	straight	straight, teardrop-shaped	straight, teardrop-shaped	straight, teardrop-shaped
Striae in 10 µm	23–25	25–30	15–18	19–27	19–29
Areolae in 10 µm	>35	25–30	24–28	27–37	34–41
Striation pattern	strongly radiate throughout	gently radiate	radiate	radiate	radiate
References	Van de Vijver et al., 2004	Bahls, 2010	Zimmermann et al., 2014	This study	
